# A scoping review of healthcare provider-led interventions for managing young children’s screen time

**DOI:** 10.1186/s12889-026-28492-w

**Published:** 2026-07-21

**Authors:** Abeer M. Alshamrani, Michael Brown, Oliver Perra, Tim Fosker

**Affiliations:** 1 School of Nursing and Midwifery, Queen’s University Belfast, Belfast, UK; 2 Prince Sultan Military College of Health Sciences, Dhahran, Saudi Arabia; 3 School of Psychology, Queen’s University Belfast, Belfast, UK

**Keywords:** Screen time, Media exposure, Smartphone use, Healthcare interventions, Caregiver education

## Abstract

**Background:**

Excessive use of screens by children has become a critical concern because of its potential impact on their health and well-being. This appears to be a global problem with many young children far exceeding international screen time guidelines. Caregivers’ interventions may play a pivotal role in managing children’s screen time. However, to develop these interventions, the key design elements that contribute to their successful implementation across different countries and cultural contexts need to be identified. Therefore, this review aims to identify the range of healthcare provider-led intervention strategies for caregivers to manage children’s screen time.

**Methods:**

A scoping literature review was conducted following the Joanna Briggs Institute (JBI) guidelines and adhering to PRISMA-ScR guidelines (Preferred Reporting Items for Systematic Reviews and Meta-Analyses extension for Scoping Reviews). Employing six databases: CINAHL, PsycINFO, ERIC, Medline, Maternity and Infant Care, and Cochrane, ten studies published between 2012 and 2023 were identified that met the review eligibility criteria.

**Results:**

Six characteristics of the interventions were systematically extracted and narratively reviewed: (1) available interventions for caregivers; (2) theoretical models used in the intervention design; (3) sociocultural considerations; (4) barriers to intervention implementation (5) facilitators to intervention implementation; and (6) implementation strategies for intervention sustainability.

**Conclusion:**

This review provides a comprehensive overview of key elements used in healthcare provider-led interventions and identifies significant gaps, including the absence of evidence from the Middle East and North Africa (MENA) region. The findings emphasise the importance of culturally informed, flexible, and caregiver-centred approaches in future intervention development and evaluation.

**Trial registration:**

The scoping review protocol was not pre-registered.

**Supplementary Information:**

The online version contains supplementary material available at https://doi.org/10.1186/s12889-026-28492-w.

## Background

Excessive screen use by children has emerged as a critical concern for their development, with implications for various facets of health and well-being [1–5]. Early childhood is a period of rapid growth and development, during which children may be highly sensitive to environmental influences, including digital media [6]. The potential health risks of excessive screen time are recognised by professional organisations such as the World Health Organisation (WHO) and the American Academy of Paediatrics (AAP), both of which have adopted a precautionary approach. Both the WHO and the AAP recommend zero screen time for children under 2 years of age, except for video chatting [7, 8]. However, many young children far exceed these guidelines [9].

Caregiver interventions have the potential to play a pivotal role in shaping children’s media habits and thus reduce excessive screen time [10]. By implementing strategies to regulate and monitor children’s screen use, caregivers can promote healthy behaviours, such as improved sleep routines, increased physical activity, and positive social interactions within the family environment [11]. Despite the growing recognition of caregiver influence on children’s media consumption, there remains a need to explore the drivers of intervention approaches and strategies that are most helpful in managing screen time in young children.

Evidence suggests that high levels of screen time among young children and caregiver difficulties managing this behaviour are international issues spanning the developed world. Increasing trends in children’s use of digital media have been observed globally, particularly in the aftermath of the COVID-19 pandemic [12]. For example, in Saudi Arabia, children watch television for approximately 2 h per day and spend about another hour using mobile media devices (3 h in total), with ownership of devices starting as early as 6 years old for tablets and 9 years old for smartphones [13–16]. Although television is the most used media device among children in Saudi Arabia (81.0%), relatively new devices, mobile phones (70.5%), and tablets (61.0%), are close competitors [17]. Similarly, in the UK the national regulator for broadcasting, telecommunications, and online safety (Ofcom) reported that seven out of ten children aged 3 to 4 years old engage in digital entertainment, with YouTube being their most-used app (87%) and tablets the most common device (75%) [18] Additionally, 42% of UK caregivers with children aged 3 to 17 years old, believe that the potential risks of social media outweigh the benefits, yet 30% of caregivers with 3–4 year-old children struggle to regulate the children’s screen time [18]. In the USA, a 2023 Statista survey found that 81% of caregivers of children aged 2–12 reported using YouTube as their primary platform [19]. In 2024, more than 60% of US teenagers owned their own cell phones [20]. By 2025, nearly 80% of caregivers reported taking steps to manage their children’s screen time, with 58% setting limits, 53% encouraging offline activities, and 34% using caregiver-control apps [21].

While the prevalence of screen use is comparable across regions worldwide, sociocultural contexts shape caregivers’ approaches to regulating screen time. In western countries, interventions often emphasise individual caregiver responsibility, structured rules, and digital literacy education [22, 23]. In contrast, in collectivist societies such as Saudi Arabia and East Asia, factors such as extended family involvement, religious guidelines, and societal norms influence parental mediation strategies [13–16].

### Rationale

The rationale for this scoping review is to bring together and assess existing evidence on healthcare-provider–led interventions aimed at managing screen time in young children. By synthesising findings from international studies, this review maps the range of intervention approaches attempted, the theoretical rationales supporting them, and the factors that influence their implementation. A Consideration of the range of approaches across different healthcare and cultural settings will support the development of recommendations for interventions tailored to the needs of families with young children globally.

### Significance

The significance of this review lies in its potential to inform policy, practice, and future research aimed at promoting healthy screen time behaviours among young children. By focusing on interventions delivered by healthcare providers, this review will clarify the nature and scope of existing approaches and highlight strategies that are both practical and acceptable within healthcare settings. Examining interventions across different countries and cultural contexts will also help identify key design elements that contribute to their attainment and consistency.

### Aim and research questions

The aim of this scoping review is to identify the range of intervention strategies delivered to caregivers to manage children’s screen time. To meet this aim, the review will answer the following questions:

- What healthcare provider-led interventions are delivered to caregivers to manage children’s screen time?
- What theoretical frameworks have been used in designing these interventions?
- What modes of delivery and evaluation methods have been used in these interventions?
- What sociocultural factors could impact the implementation of children’s screen time interventions?

## Methods

Scoping reviews are used to systematically map existing literature on a topic, identifying key concepts, theoretical frameworks, sources of evidence, and research gaps [24]. This approach was selected because the questions aim to explore the breadth of the literature and discuss various types of interventions and their characteristics. This review was conducted following Joanna Briggs Institute (JBI) guidelines [24], which do not require formal critical appraisal tools, and adhere to the PRISMA-ScR statement [25].

### Search strategy

An initial scoping search was conducted in the Cochrane and Medline databases to identify keywords (words and phrases) in titles and abstracts that would inform the final search strategy. The formal keyword search was conducted on 10th March 2025 and re-run on 20th January 2026 via six electronic databases: CINAHL, PsycINFO, ERIC, Medline, Maternity and Infant Care, and Cochrane (see Table 1 for keywords). All the retrieved articles from the selected databases were imported into Covidence^®^, a systematic review tool, separately per database. Covidence^®^ was used to eliminate duplicate studies, manage references electronically, and facilitate the screening process. Aes used during the review.ppendix 1 contains the detailed full search strategi

**Table 1:** Identified keyword search terms

Search Key term	
	Caregiver* OR Parent* OR Mother* OR Father
AND	Preschool* OR Child* OR Infant OR Toddler* Paediatric
AND	Screen time OR Media Exposure OR Smartphone
AND	Training* OR Program* OR Intervention* OR Education* OR Experiment* OR Randomised*

The asterisk (*) indicates truncation. Boolean operators (AND/OR) were used to combine search terms in the database search strategy. *

### Eligibility criteria

Eligibility criteria were linked directly to the research aim and questions using the Population Concept and Context (PCC) framework [26].

### Inclusion Criteria

Population:

1. Intervention was delivered to caregivers of children aged birth to 5 years.

Concept:

1. Intervention delivered to caregivers aimed at reducing/managing screen time in children aged birth to 5 years.
2. Interventions target caregivers as the primary agents of change.
3. Studies reporting quantitative data as the review aims to identify and map healthcare provider-led interventions with measurable outcomes, which are best captured through quantitative designs [24].
4. Studies published in English and peer-reviewed journals.
5. Studies that evaluate or consider the 2011 screen time recommendations for children as outlined by the American Academy of Paediatrics (AAP), which reflected the emergence of new screen-based technologies, and is taken to represent a relevant starting point for analysing evidence of contemporary interventions.

Context:

1. Healthcare provider-led interventions are defined as those in which a trained health professional, for example, a nurse or allied health worker, has a role in initiating, designing, delivering, supervising, or clinically guiding the intervention, regardless of delivery mode or setting.
2. The intervention was delivered/introduced initially by healthcare providers in clinical settings, such as hospitals, outpatient clinics, and other healthcare facilities, where they served as the primary initial point of delivery. For app-based interventions, healthcare providers in these settings facilitated, guided, or simply provided caregivers with access to the app, even though caregivers may subsequently have used the app independently and remotely.
3. The intervention initially consists of a primary session during which caregivers engage directly with program deliverers. Follow-up sessions are defined as any subsequent interactions occurring after the primary session, specifically designed to provide ongoing support to assess screen time behavioural changes. These follow-ups are distinct from the initial session and are focused on continued engagement rather than the initial delivery of the intervention.

### Exclusion criteria

1. Studies focusing solely on children with developmental disorders or medical conditions. such as autism spectrum disorder (ASD), attention-deficit/hyperactivity disorder (ADHD), intellectual disability, specific learning disorders (e.g., dyslexia, dyscalculia), and speech and language disorders.
2. Interventions conducted in school settings or at home as research settings.
3. Studies published before the 2011 screen time recommendations for children as outlined by the American Academy of Paediatrics (AAP).
4. Interventions primarily that target schools, healthcare providers, or other external agents without a primary focus on caregivers’ involvement.
5. Conference abstracts and grey literature, including government reports, policy statements, and unpublished research.
6. Studies with designs other than quantitative research, including observational studies, qualitative studies, case studies, posters and oral presentations, preprints, dissertations, and reviews (systematic, scoping, and meta-analysis).

### Selection of the source of evidence

All reviewers worked in parallel using Covidence, where the predefined inclusion and exclusion criteria were embedded, colour-coded and accessible to the full team. A review protocol was developed a priori to guide the review process; however, it was not formally registered. After duplicate removal by (AA), two reviewers (AA and OP) independently screened titles and abstracts, followed by independent full-text screening using the same criteria. A random 20% sample was independently verified by a third reviewer (TF) to ensure consistency in screening decisions. Throughout the screening for each stage Covidence automatically flagged conflicts; those discrepancies were resolved through discussion and refinement of the criteria. Inter-rater reliability was assessed within the platform, with agreement improving after criteria refinement, indicating consistent application of the screening criteria.

### Data extraction

The data extraction form was piloted on a sample of studies prior to full use to ensure alignment with the review questions and was refined accordingly. Data extraction was conducted independently by the reviewers (AA & OP), and the results were then compared to verify consistency in the extracted information. The data extraction included study characteristics such as authorship, publication year, and study design; sample sizes; intervention details (description, who delivered it [e.g., healthcare providers, parents], duration, and frequency of sessions); and other pertinent information. Outcomes, including primary and secondary outcomes, were also extracted. Key findings were summarised based on reported inferential statistics (see Table 3).

### Data synthesis and presentation

Initially, a narrative descriptive summary of the characteristics of the included studies was developed. Then, a structured data-extraction table was created to capture key information relevant to the review questions, including study details, theoretical underpinnings, intervention descriptions, and screen time-related measures and results. Finally, the extracted data were synthesised narratively to identify patterns across studies. A narrative synthesis was used to identify and discuss the range of characteristics of healthcare-provider–led interventions rather than to evaluate their effectiveness.

## Results

**Fig. 1:**
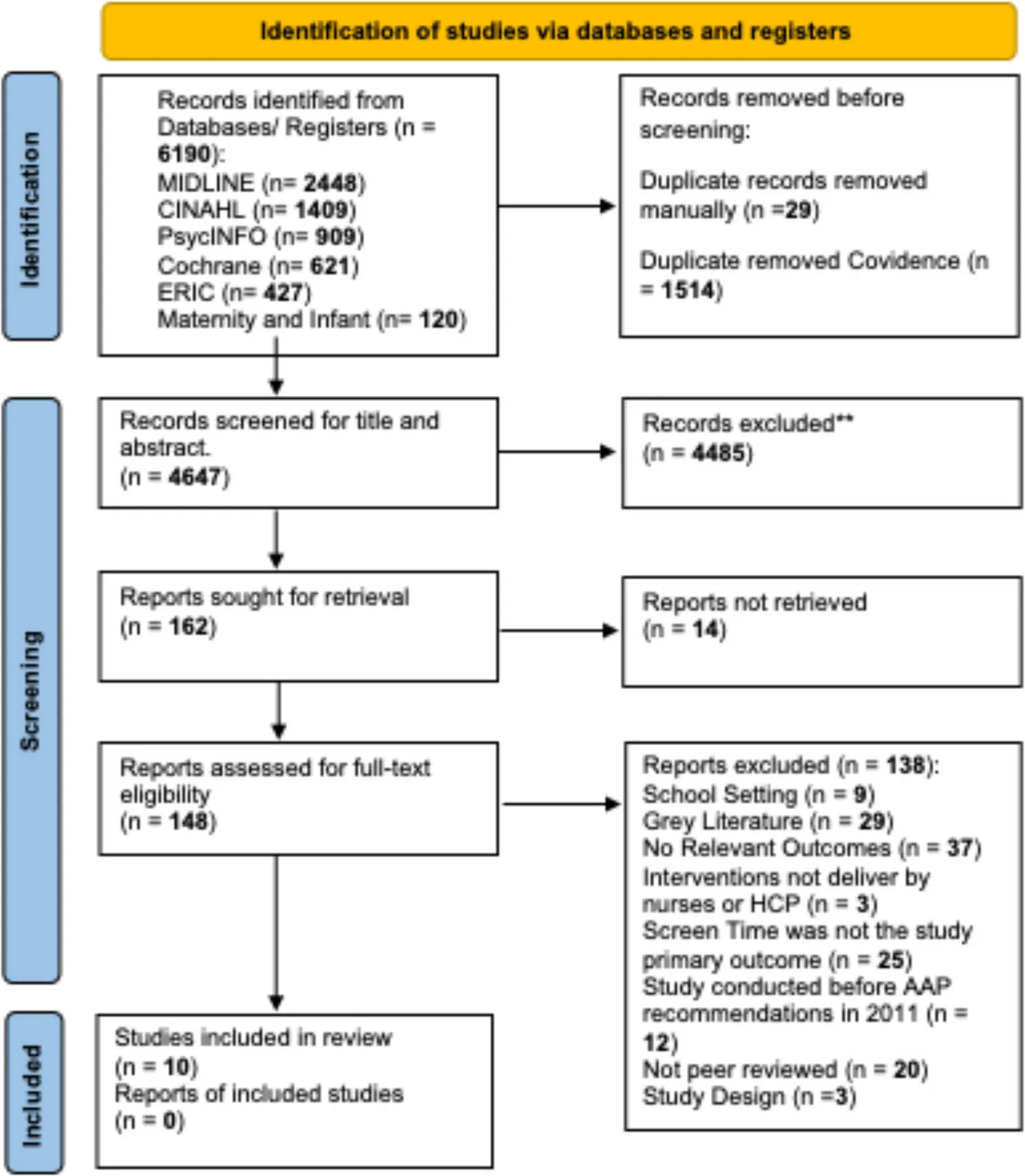
PRISMA flow diagram

The PRISMA flow diagram (Fig. 1) summarises the study selection process. A total of 6,190 records were identified through database searching and other sources. After removing duplicates, 4,647 records were screened by title and abstract. Of these, 148 full-text articles were assessed for eligibility, and 138 were excluded for the following reasons: conducted in school settings (*n* = 9), grey literature (*n* = 29), interventions not delivered by healthcare providers or nurses (*n* = 3), screen time not the primary outcome (*n* = 25), conducted before the 2011 AAP screen time recommendations (*n* = 12), published in non–peer-reviewed journals (*n* = 20), inappropriate study design (*n* = 3), and no relevant outcomes (*n* = 37). Ten studies met the inclusion criteria and were included in the final review.

Flow diagram showing the number of records identified, screened, excluded, and included in the review.

### Study characteristics

Of the 10 included studies, five were conducted in the USA [27–31], one was conducted in Sweden [32], one in Australia [33], one in India [34], one in Malaysia [35], and one in Thailand [36] (see Table 2).

The populations studied included diverse demographics, with some studies focusing on infants and others on caregivers, and sample sizes varying from 17 participants [30] to a large-scale trial of 552 participants [32] (see Table 3). Randomised controlled trials were the most common study design, comprising seven studies [27–29, 32–35]. The remaining three studies utilised quasi-experimental designs [30, 31, 36].

The studies demonstrated considerable variability in the types of interventions that were evaluated, including mHealth and digital approaches such as mobile applications [32], short text messaging [30], and communication via social platforms [35], alongside in-person interventions, including individual face-to-face sessions [27, 29, 31, 34, 36] and group discussions [33].

The key findings across the included studies demonstrated significant improvements in children’s screen time behaviours, caregivers’ healthy media practices, and/or caregivers’ screen time management. However, three studies did not yield statistically significant results, despite the interventions having a positive influence on caregivers’ knowledge or overall child screen time practices [29, 30]. Characteristics of the studies and interventions are summarised in Tables 2 and 3.

**Table 2:** Study characteristics

Authors	Country	Study Design
Adams et al. [27].	USA	RCT (two-arm)
Alexandrou et al. [32].	Sweden	RCT (two-arm)
Beck et al. [28].	USA	RCT (two-arm)
Birken et al. [29].	USA	RCT (two-arm)
Brown et al. [30].	USA	Quasi-experimental (one-arm)
Delisle Nyström et al. [33].	Australia	Cluster RCT (two-arm)
Poonia et al. [34].	India	RCT (two-arm)
Raj et al. [35].	Malaysia	RCT (two-arm)
Tucker et al. [31].	USA	Quasi-experimental (two-arm)
Boonmun et al. [36].	Thailand	Quasi-experimental (two-arm)

Study characteristics including authors, publication year, country, and study design for all included interventions

*RCT*  Randomised Controlled Trial

**Table 3:** Intervention characteristics

No	Authors	Sample	Theoretical Background	Intervention Description (Name, Delivery Mode, Content)	ST- Related Results
1.	Adams et al. [27].	*n* = 279 Intervention *n* = 140 Control *n* = 139	Responsive Parenting (RP) Theory; AAP Guidelines	o Intervention: INSIGHT RP o Delivery Mode: Nurse-delivered at research centre (6 contacts: 3–40 weeks, 1–2 years) including messages on parenting practices. o Content: Age-appropriate messages on minimising screen time per AAP recommendations	Intervention group showed significantly lower daily ST at all time points vs. control
2.	Alexandrou et al. [32].	*n* = 552 Intervention *n* = 277. Control *n* = 275.	Social Cognitive Theory (observational learning, modelling)	o Intervention: MINIS- TOP 2.0 app o Delivery Mode: mHealth smartphone app; integrated into primary child healthcare; o Content: 13 biweekly themes covering diet, physical activity, ST, sleep, and dental care	The intervention group showed significantly lower daily ST at follow-up vs. control
3.	Beck et al. [28].	*n* = 96 Intervention *n* = 48. Control *n* = 48.	Information-Motivation-Behavioural Skills model (IMB)	o Intervention: Futuros Fuertes (Strong Futures). o Delivery Mode: Individual health education post well-child visits; bilingual educators; biweekly text messages (2 weeks–15 months) o Content: 4 themes: diet, physical activity, ST, and sleep; addressed ST impact on infant development from age 2 months	Intervention group showed significantly lower daily ST vs. control at 6, 12, and 15 months
4.	Birken et al. [29].	*n* = 160 Intervention *n* = 64. Control *n* = 68.	Goal setting, positive reinforcement, monitoring, and cognitive restructuring	o Intervention: Behavioural counselling intervention o Delivery Mode: In-person 10-min sessions by dietitians at child immunisation clinic o Content: ST health impacts, strategies to reduce ST.	No significant reduction in ST on weekdays or weekends vs. control
5.	Brown et al. [30].	*n* = 17 Intervention *n* = 17.	Social Cognitive Theory (role modelling, environmental structuring)	o Intervention: SMS Parent Action Intervention o Delivery Mode: Text messages from paediatrician/dietitian over 5 weeks (3 messages/week; 15 total) o Content: 5 themes: sleep, nutrition, ST, physical activity, healthy beverages; ST addressed per AAP recommendations	No significant reduction in ST post-intervention
6.	Delisle Nyström et al. [33].	*n* = 492 Intervention *n* = 249. Control *n* = 243.	Anticipatory Guidance Framework (parenting skills)	o Intervention: INFANT (Melbourne Infant Feeding Activity and Nutrition Trial). o Delivery Mode: Six group sessions over 15 months at maternal and child health centre; supplementary take-home materials o Content: ST recommendations, developmental impact, alternative activities, and strategies to limit ST per AAP recommendations.	Intervention group showed significantly improved maternal TV viewing knowledge and reduced children’s TV viewing at 2-year and 3.5-year follow-ups vs. control
7.	Poonia et al. [34].	*n* = 120 Intervention *n* = 60. Control *n* = 60.	Anticipatory Guidance Framework (parenting skills)	o Intervention: Parental education session o Delivery Mode: Single 30-min in-person counselling session at immunisation clinic o Content: Age-appropriate responsive parenting skills, limiting ST exposure, modifying parental media habits per AAP recommendations	Intervention group showed significantly lower daily ST vs. control
8.	Raj et al. [35].	*n* = 360 Intervention *n* = 180. Control *n* = 180.	Social Cognitive Theory (knowledge, goal setting, self-efficacy, outcome expectation, observational learning, problem-solving)	o Intervention: Stop and Play o Delivery Mode: Remote delivery via WhatsApp o Content: Educational videos, infographics, problem-solving sessions, and success stories targeting ST reduction and physical activity promotion	Intervention group showed significantly lower daily ST vs. control
9.	Tucker et al. [31].	*n* = 165 Intervention *n* = 93 Control *n* = 72	Behavioural Change Models; Primary Care Integration	o Intervention: “We Are for Children” program. o Delivery Mode: Physician-family conversations at well-child visits; four monthly dietitian sessions over 6 months; optional social worker counselling. Content: Healthy messaging, feeding practices, nutrition education, and goal setting	Intervention group showed significantly lower daily ST vs. control
10.	Boonmun et al. [36].	*n* = 70 Intervention *n* = 35 Control *n* = 35	Ajzen’s Theory of Planned Behaviour (TPB).	o Intervention: Parents’ Screen Time Reduction for Preschool Children Program (PSTRPP). o Delivery Mode: RN-delivered at childcare centres; 3 sessions over 2 weeks (3 h/session) o Content: Building parental attitudes, perceived norms, perceived control, and intentions to reduce children’s ST	Intervention group showed significantly lower daily ST vs. control

Intervention characteristics including population, theoretical background, delivery mode, content, screen time measures, and screen time–related outcomes for all included studies

*AAP *American Academy of Paediatrics, *RP* Responsive Parenting

### Narrative synthesis of the findings

The results were narratively synthesised, and the six themes outlined below emerged from the analysis.

### Available interventions for caregivers

Face-to-face interventions provide caregivers with direct guidance on managing children’s screen time [27, 29, 31, 33, 34, 36]. These interventions frequently included group discussions and role-playing exercises. However, some studies reported challenges with caregiver participation, citing time constraints and logistical difficulties with delivery and access as key barriers [27, 35]. Digital interventions, including mobile apps and web-based programmes, were favoured by participants for their convenience and scalability, with 3 of the 10 studies utilising digital formats. Some studies found that digital tools with real-time feedback and goal setting features supported caregiver engagement and adherence to screen time limits [28, 30, 32]. However, accessibility issues, such as limited digital skills or poor internet connection, posed challenges in some cases [32]. Finally, hybrid interventions, which combine face-to-face sessions with digital reinforcement, balanced accessibility with structured support and were reported to support healthier behaviours in children. Study findings suggested that an initial in-person session followed by app-based reminders or text messages can help caregivers maintain engagement and apply their learning [28].

### Theoretical models used in the intervention design

Theoretical frameworks informed all ten interventions. While Social Cognitive Theory informed three interventions in this review, each applied different SCT components. Alexandrou [32] emphasised observational learning and caregiver modelling; Raj et al. [35] incorporated a broader set of SCT constructs, including goal setting, self-efficacy, outcome expectations, and problem-solving; while Brown et al. [30] focused on caregiver role modelling and environmental structuring. Another three interventions used other behaviour change models, including the Information-Motivation-Behavioural Skills model [28], behavioural health theories [31], and programmes structured around behavioural techniques such as goal setting, reinforcement, and monitoring [29]. Three interventions were grounded in parenting-focused frameworks. The INSIGHT study applied a Responsive Parenting approach, using structured guidance to help caregivers respond appropriately to infant cues and establish healthy routines [27]. Two interventions used anticipatory guidance frameworks, providing caregivers with developmentally tailored advice on managing emerging behaviours, including screen time practices [33, 34]. One intervention applied the Theory of Planned Behaviour to target caregiver attitudes, perceived behavioural control, and social norms related to managing children’s screen time practices [36].

### Sociocultural considerations

Across the ten studies, multiple sociocultural factors were identified. Cultural household practices were reported in several studies, including eating in front of screens and the presence of televisions or other digital devices in children’s bedrooms [29, 35]. Caregiver beliefs also shaped screen use patterns, with Raj et al. [35] noting that some caregivers viewed screen time as beneficial for language development, reflecting broader caregiver beliefs about educational media. Family routines and roles further influenced engagement; for example, mothers in the Futuros Fuertes intervention used messages to encourage partners to adopt healthier practices, reflecting gendered caregiving dynamics in which screen time management fell primarily to mothers. Some caregivers reported already following similar routines [28, 30]. Maternal characteristics such as higher pre-pregnancy BMI, younger age, and greater television-viewing knowledge were associated with children’s viewing patterns, suggesting that individual caregiver profiles may moderate intervention reach [27, 33]. Linguistic diversity affected accessibility, prompting the translation of digital content for families with foreign backgrounds [32]. Additional factors included caregiver modelling of screen use [36] and cultural tailoring processes, such as pretesting materials with local families [28, 35]. The included studies also reflected variation in how screen time responsibility was framed across cultural contexts. Studies from western settings framed screen time regulation as an individual parental responsibility, with interventions targeting mother-child dyads [27] or individual parents in primary care [29, 31–33] In contrast, one study noted that the Malaysian context involved collective family structures, including bed-sharing and shared household practices [35]. Another study found through qualitative data that Latino parents shared intervention content with partners and other family members, collectively negotiating screen-time decisions rather than managing them individually [28].

### Barriers to intervention implementation

Four studies reported caregiver and environmental barriers, including caregivers’ difficulty in reducing children’s screen time, varying interpretations of intervention messages, limited living space in low-income households, and contextual disruptions such as pandemic-related restrictions and seasonal constraints [27, 28, 35, 36]. In addition, caregiver attitudes influenced engagement, with some caregivers perceiving screen time as beneficial for language development, which shaped how they applied intervention guidance [35]. Restricted generalisability was reported in five studies with narrow or demographically specific samples, low participation rates, or recruitment challenges [28, 30, 31, 35, 36]. Several interventions showed limited or non-significant effects on primary outcomes such as screen time, physical activity, or BMI, often due to the shortness of intervention duration, low intensity, or short follow-up periods [27, 29, 31, 33, 34]. Additional barriers included a lack of standardised screen time measurement tools and the evolving nature of screen-based devices in terms of their types, functions, and patterns of use [29, 33].

### Facilitators to intervention implementation

Interventions that utilised theoretical or conceptual frameworks (e.g., TPB, SCT, responsive parenting, anticipatory guidance) may strengthen caregiver knowledge, skills, and self-efficacy [27, 33–36]. Seven studies highlighted the advantages of early, developmentally timed delivery, often embedded in routine primary care or child health services, which provided consistent contact and credible guidance [27, 29, 31–33]. Six studies identified multicomponent or structured approaches (clinician counselling, dietitian support, group discussions, or repeated reinforcement) as facilitators of changes in caregiver feeding practices, screen time, and parent–child interaction [31, 34, 36]. Digital delivery methods, including apps, WhatsApp videos, and tailored text messages, facilitated flexible access, ongoing reminders, and two-way communication [28, 30, 32, 35]. Caregiver engagement was further supported through personalisation, education by local healthcare professionals, multilingual adaptation, and culturally relevant, low-cost activity suggestions, which enhanced engagement and acceptability across four studies [28, 30, 32, 35].

### Implementation strategies for intervention sustainability

A small number of studies reported strategies related to sustaining interventions over time. In the area of planning and evaluation, two studies highlighted the need for long term follow-up to determine whether intervention effects are maintained [33, 35]. Raj et al. [35], also emphasised pre-implementation planning, including ensuring adequate staffing and training, and examining the intervention’s applicability across diverse socioeconomic groups to support broader use. In addition, one study reported that exploring aspects of implementation through qualitative interviews with families, healthcare professionals, and stakeholders may support future adoption within primary child healthcare [32].

## Discussion

This scoping review provides a consolidated understanding of how healthcare provider-led interventions have been designed and delivered to caregivers, while also identifying common sociocultural influences and the potential facilitators and barriers involved in implementing children’s screen time interventions.

Across the included studies, three broad insights stand out. First, delivery mode shapes both engagement and feasibility: face-to-face interventions offer relational depth but are constrained by practical barriers, while digital formats increase accessibility yet introduce inequities tied to digital readiness. Second, theoretical grounding, particularly through Social Cognitive Theory, the Theory of Planned Behaviour, behavioural models, and parenting-focused frameworks, strengthens the application of behaviour change strategies. Third, sociocultural norms, caregiver beliefs, and household structures may influence how caregivers interpret and apply guidance. Notably, definitions of screen time varied across the included studies, encompassing television viewing, mobile device use, and total sedentary exposure, which may limit direct comparisons.

Regarding delivery methods, face-to-face delivery remains a strength of many interventions [27, 29, 31, 33, 34, 36]. In-person formats consistently supported caregiver engagement by providing personalised guidance, opportunities for modelling, and relational immediacy [37]. These mechanisms align with broader behavioural evidence showing that trust, rapport, and interactive learning all enhance motivation and self-efficacy [38]. Even brief counselling during routine paediatric visits [34] has been shown to have the potential to shift caregiver practices, underscoring the value of embedding guidance within existing healthcare relationships [39]. However, this review reveals multiple barriers to completing a screen time intervention that are common to all caregiver-mediated interventions, namely time pressures and contextual disruptions, such as logistical restrictions and seasonal constraints [27, 28, 35, 36]. These constraints were not limited to any single demographic group; they appeared across socioeconomic contexts, suggesting that traditional in-person programmes often demand more time and stability than many families can realistically provide [40]. High attrition rates in multi-session programmes illustrate the difficulty of sustaining participation when interventions are rigid or resource-intensive [41].

Digital delivery emerged as a response to the above challenges [30, 32, 35]. These findings align with the broader literature, which indicates that online platforms, apps, and remote communication tools offer flexibility, scalability, and reduced burden on families [42, 43]. Features such as real-time feedback, goal setting, and automated reminders support engagement and align with behaviour change principles [44, 45]. Yet digital interventions have also encountered few inequities [32], particularly among families with limited digital literacy, unstable internet access, or low confidence in navigating technology, thereby hindering full participation [45]. Another finding that stands out from this review is that hybrid models combining initial face-to-face contact with digital follow-up are feasible [28]. This approach appears to offer a promising middle ground [43]. Although only one screen time intervention used this format directly [28], evidence from broader parenting and mental health programmes suggests that blended approaches can preserve relational benefits while reducing logistical burden [46, 47].

Interventions grounded in behavioural theory demonstrated clearer mechanisms and more coherent strategies than those relying on general guidance or non-theoretical approaches [11]. In this review, Social Cognitive Theory [30, 32, 35] and parenting-focused frameworks [27, 33, 34] were the most commonly applied approaches, informing techniques such as goal setting, self-monitoring, reinforcement, and shifts in caregiver attitudes and perceived control. These approaches have been similarly applied in other literature to influence caregiver attitudes [48], and meta-analytic evidence suggests that theory-driven interventions produce more sustained behaviour change than generic educational approaches [49]. The depth of theoretical application mattered [11]. Evidence from broader intervention research suggests that interventions that explicitly map components to behaviour change techniques, such as planning, feedback, and behaviour substitution, tend to achieve stronger outcomes than those that reference theory only superficially [50].

In this review, several interventions applied theoretical constructs in a detailed and explicit way, for example, Alexandrou et al. [32], Raj et al. [35], and Brown et al. [30] operationalised specific components of Social Cognitive Theory to guide modelling, goal- setting, self-efficacy, and environmental structuring, while Boonmun et al. [36] used Theory of Planned Behaviour constructs to target caregiver attitudes and perceived control. By contrast, interventions drawing on the IMB model [28], behavioural health theories [31], or general behavioural techniques [29] referenced theory more broadly without detailing how theoretical constructs informed the selection of specific strategies. Parenting-focused approaches added an additional layer by emphasising parent–child interaction and the home environment, which aligns with evidence showing that parent-involved interventions are central to reducing preschoolers’ screen time [51]. These principles reflect the socioecological nature of screen use, where children’s behaviours are shaped by caregivers’ routines, household norms, and broader cultural expectations. This review highlights the need for future interventions to explain how their chosen theoretical constructs translate into specific behaviour change strategies, rather than referencing theory in general terms.

Sociocultural factors shaped the feasibility of the intervention in ways that went beyond simple barrier identification. Where household routines normalised screen exposure and caregivers viewed screens as educationally beneficial, reduction messaging met resistance [28, 35]. Cultural tailoring, including pretesting materials with local families, improved acceptability by reflecting caregivers’ real-world contexts [28, 35].

Screen time decisions are rarely made by individual caregivers alone. Partner engagement influenced how messages were received [28], reflecting gendered caregiving dynamics in which mothers primarily managed screen time. Caregiver modelling emerged as a key behavioural mechanism linking caregivers’ habits to children’s exposure [36]. Maternal characteristics, including age, BMI, and screen-related knowledge, were associated with children’s viewing patterns [27, 33], suggesting that individual caregiver profiles may moderate intervention reach. Linguistic diversity required adaptation beyond content alone, including translating materials to ensure basic access for families with foreign backgrounds [32].

These findings align with broader evidence that family socioeconomic and contextual factors shape how caregivers manage children’s screen time. Caregivers’ approaches to screen use are influenced by family routines, neighbourhood conditions, and broader structural constraints rather than universal guidance alone [52, 53]. Socioeconomic pressures restrict families’ capacity to reduce screen time [54]. These contextual factors suggest that future feasibility work must move beyond individual behaviour-change approaches to examine the broader social and environmental conditions that shape caregivers’ decision-making.

Across the studies reviewed, personalised guidance appeared to facilitate caregiver engagement [28, 30, 32, 35]. This aligns with the broader literature, which shows that personalised guidance strengthens caregivers’ self-efficacy in managing children’s screen time [55–57]. However, personalisation alone was insufficient when caregivers viewed screens as educational, convenient, and harmless [35], which is consistent with the broader literature, particularly where screens are already deeply embedded in daily routines [55]. Similarly, flexible delivery helped families navigate competing demands [58], yet it could not overcome logistical barriers such as limited safe play spaces or a lack of childcare support [55, 57]. Digital tools enhanced monitoring and consistency but only functioned as facilitators when caregivers possessed adequate digital literacy [51]. Finally, early, developmentally timed delivery by a trusted healthcare provider was a recurring theme across the included studies [27, 29, 31–33]. This aligns with evidence from a meta-analysis of 204 studies, which suggests that screen time interventions targeting younger children are associated with larger treatment effects, and that healthcare provider delivery incorporating goal setting and feedback strategies further enhances effectiveness [49]. Overall, these mechanisms highlight that interventions may work better when they align with caregivers’ realities and do not assume ideal conditions that many families cannot meet [59].

Sustainability received limited attention across the included interventions, indicating that long-term maintenance remains an underdeveloped area in early childhood screen time research. The few studies that addressed it highlighted two priorities: (1) the need for structured follow-up to assess whether behaviour change persists, particularly given evidence that effects often decline once support is removed [60]; and (2) the importance of practical planning for staffing, training, and socioeconomic applicability to enable wider adoption. Qualitative insights from families and practitioners further underscored the value of understanding real-world implementation conditions. In general, the evidence shows that long-term maintenance improves when interventions are embedded within existing systems, supported by ongoing reinforcement, and responsive to the daily realities of family life [54, 55, 61].

## Strengths and limitations

This scoping review demonstrates several methodological strengths, following established scoping review frameworks to ensure transparency and reproducibility. A comprehensive multi-database search strategy was employed, and dual independent screening was conducted throughout, minimising selection bias. The inclusion of studies from multiple countries further enhances the international applicability of the findings. However, several limitations must be acknowledged. Restricting the review to English-language studies only may have excluded relevant evidence from non-English-speaking contexts. In addition, excluding other study designs may limit insight into the contextual and experiential aspects of implementation. Finally, rapid technological changes over the period governed by the review may have influenced how screen exposure was measured and reported in different studies.

## Conclusion

This review suggests that healthcare provider-led interventions for managing children’s screen time are most promising when they are theoretically grounded, culturally responsive, and flexible enough to accommodate the realities of family life. Interventions that combine theoretical grounding, personalised guidance, hybrid delivery, and introduction by healthcare providers during the child’s early developmental period appear particularly promising. However, persistent barriers, including low participation rates, recruitment challenges, digital inequities, and caregivers’ attitudes, highlight the need for adaptable, context-sensitive approaches. Structured follow-up sessions to support sustainability are particularly pertinent to future work. Notably, no studies from the Middle East and North Africa (MENA) region were identified, limiting the review’s ability to capture caregivers’ experiences in those cultural contexts. Future work should continue to refine support strategies, with particular attention to sustainability, cultural relevance, and equitable access.

## Supplementary Information

Supplementary Material 1: Appendix 1 Full Search Strategies.

## Data Availability

All data used in this review are derived from previously published studies and are available in the manuscript.

## Abbreviations

TPB: Ajzen’s Theory of Planned Behaviour
AAP: American Academy of Paediatrics
ADHD: Attention-Deficit/Hyperactivity Disorder
ASD: Autism Spectrum Disorder
BMI: Body Mass Index
COVID-19: Coronavirus Disease 2019
IMB: Information-Motivation-Behavioural Skills
JBI: Joanna Briggs Institute
Ofcom: National Regulator for Broadcasting, Telecommunications, And Online Safety
PSTRPP: Parents’ Screen Time Reduction for Preschool Children Program
PCC: Population Concept and Context
RP: Responsive Parenting
SMS: Short Messaging System
SCT: Social Cognitive Theory
WHO: World Health Organization
The PRISMA-Scr: The Preferred Reporting Items for Systematic Reviews and Meta-Analyses Extension for Scoping Reviews
