# Supplementary material for: A scoping review of healthcare provider-led interventions for managing young children’s screen time
Source: BMC Public Health. 2026 Jul 21;26:2984. doi: 10.1186/s12889-026-28492-w (PMC13673749; doi:10.1186/s12889-026-28492-w)
Supplement: Supplementary file 1 [file 12889_2026_28492_MOESM1_ESM.docx]

1. **Database:** Maternity & Infant Care Database

| **#** | **Query** | **Results from 20 Jan 2026** |
| --- | --- | --- |
| 1 | Caregiver* | 3,111 |
| 2 | Parent* | 28,783 |
| 3 | Mother* | 65,361 |
| 4 | Father* | 6,025 |
| 5 | 1 or 2 or 3 or 4 | 88,430 |
| 6 | Preschool* | 1,413 |
| 7 | Child* | 82,875 |
| 8 | Infant* | 112,911 |
| 9 | Toddlers* | 959 |
| 10 | pediatric* | 8,021 |
| 11 | 6 or 7 or 8 or 9 or 10 | 164,020 |
| 12 | Screen Time | 72 |
| 13 | Media Exposure | 154 |
| 14 | SmartPhone* | 262 |
| 15 | Training* | 13,138 |
| 16 | Program* | 25,865 |
| 17 | Intervention* | 39,617 |
| 18 | Education* | 33,188 |
| 19 | Experiment* | 4,605 |
| 20 | Randomized | 15,783 |
| 21 | 12 or 13 or 14 | 480 |
| 22 | 15 or 16 or 17 or 18 or 19 or 20 | 98,621 |
| 23 | 5 and 11 and 21 and 22 | 120 |

<https://qub.idm.oclc.org/login?url=https://ovidsp.ovid.com/ovidweb.cgi?T=JS&NEWS=N&PAGE=main&SHAREDSEARCHID=6Y2mnIhf7P323ysIb49QnZb76gUKalz7vDpjgQraFI72zvtCCfmn08gtzZtbYmSee>

2. **Database:** Ovid MEDLINE(R)

| **#** | **Query** | **Results from 20 Jan 2026** |
| --- | --- | --- |
| 1 | Caregiver* | 136,808 |
| 2 | Parent* | 628,315 |
| 3 | Mother* | 317,022 |
| 4 | Father* | 60,377 |
| 5 | 1 or 2 or 3 or 4 | 1,008,007 |
| 6 | Preschool* | 1,069,765 |
| 7 | Child* | 3,026,730 |
| 8 | Infant* | 1,499,711 |
| 9 | Toddlers* | 11,724 |
| 10 | P?ediatric. | 550,953 |
| 11 | 6 or 7 or 8 or 9 or 10 | 3,753,418 |
| 12 | Screen Time | 6,783 |
| 13 | Media Exposure | 2,359 |
| 14 | SmartPhone* | 36,196 |
| 15 | 12 or 13 or 14 | 44,769 |
| 16 | Training* | 746,889 |
| 17 | Program* | 1,418,349 |
| 18 | Intervention* | 1,789,499 |
| 19 | Education | 1,218,429 |
| 20 | Experiment* | 3,095,165 |
| 21 | Randomi#ed | 1,241,188 |
| 22 | 16 or 17 or 18 or 19 or 20 or 21 | 7,797,422 |
| 23 | 5 and 11 and 15 and 22 | 2,448 |

<https://qub.idm.oclc.org/login?url=https://ovidsp.ovid.com/ovidweb.cgi?T=JS&NEWS=N&PAGE=main&SHAREDSEARCHID=3uhw2kYRDiqHAqsdswhpnLu4sQzGiE70UawfDcXLTOx6n2O3olCzOpkUMRIDB7akZ>

3. **Database:** APA PsycInfo

| **#** | **Query** | **Results from 20 Jan 2026** |
| --- | --- | --- |
| 1 | Caregivers/ or Caregiver* | 88,240 |
| 2 | Parent* | 380,816 |
| 3 | Mother* | 164,661 |
| 4 | Father* | 60,146 |
| 5 | 1 or 2 or 3 or 4 | 542,193 |
| 6 | Preschool* | 124,190 |
| 7 | Child* | 980,357 |
| 8 | Infant* | 137,854 |
| 9 | Toddlers* | 9,835 |
| 10 | Pediatric/ or Pediatrics | 41,032 |
| 11 | 6 or 7 or 8 or 9 or 10 | 1,040,025 |
| 12 | Screen Time | 2,952 |
| 13 | Media Exposure | 4,340 |
| 14 | Smartphones/ or Smartphone* | 10,862 |
| 15 | 12 or 13 or 14 | 17,682 |
| 16 | Training* | 369,769 |
| 17 | Program* | 548,255 |
| 18 | Intervention* | 592,462 |
| 19 | Education* | 814,483 |
| 20 | Experiment* | 612,914 |
| 21 | Randomized | 115,522 |
| 22 | 16 or 17 or 18 or 19 or 20 or 21 | 2,203,081 |
| 23 | 5 and 11 and 15 and 22 | 1,165 |

<https://qub.idm.oclc.org/login?url=https://ovidsp.ovid.com/ovidweb.cgi?T=JS&NEWS=N&PAGE=main&SHAREDSEARCHID=1KICJx0kaNvNyQcBwkyjCnI0HwsjdH3X0iCLwBSijJfJitjJTxhpeQscGIue32y1X>

4. **Database:** Cochrane

| # | **Query** | **Results from 20 Jan 2026** |
| --- | --- | --- |
| 1 | Caregiver* | 25640 |
| 2 | Parent* | 69020 |
| 3 | Mother* | 29634 |
| 4 | Father* | 2756 |
| 5 | #1 or #2 or #3 or #4 | 111478 |
| 6 | Preschool* | 52082 |
| 7 | Child* | 248409 |
| 8 | Infant* | 83897 |
| 9 | Toddler* | 3026 |
| 10 | Pediatric | 73496 |
| 11 | #6 or #7 or #8 or #9 or #10 | 291766 |
| 12 | "Screen Time" | 887 |
| 13 | "Media exposure" | 114 |
| 14 | "Smart Phone" | 1332 |
| 15 | #12 or #13 or #14 | 2318 |
| 16 | Training* | 160752 |
| 17 | Program* | 204425 |
| 18 | Intervention* | 740637 |
| 19 | Education* | 147055 |
| 20 | Experiment* | 227373 |
| 21 | Randomized | 1396134 |
| 22 | #16 or #17 or #18 or #19 or #20 or #21 | 1775854 |
| 23 | #5 and #11 and #15 and #22 | 621 |

<https://www-cochranelibrary-com.qub.idm.oclc.org/advanced-search/search-manager?search=7404372>

5. **Database:** ERIC

| # | **Query** | **Results from 20 Jan 2026** |
| --- | --- | --- |
| 1 | Caregiver* | 16,206 |
| 2 | Parent* | 169,134 |
| 3 | Mother* | 44,040 |
| 4 | Father* | 23,640 |
| 5 | #1 or #2 or #3 or #4 | 189,502 |
| 6 | Preschool* | 67,230 |
| 7 | Child* | 425,685 |
| 8 | Infant* | 23,430 |
| 9 | Toddler* | 36,752 |
| 10 | P#ediatric | 2,624 |
| 11 | #6 or #7 or #8 or #9 or #10 | 432,979 |
| 12 | Screen Time | 520 |
| 13 | Media exposure | 2,301 |
| 14 | SmartPhone | 2,286 |
| 15 | #12 or #13 or #14 | 5,010 |
| 16 | Training* | 255,859 |
| 17 | Program* | 680,464 |
| 18 | Intervention* | 126,026 |
| 19 | Education* | 1,713,993 |
| 20 | Experiment* | 127,445 |
| 21 | Randomi?ed | 10,569 |
| 22 | #16 or #17 or #18 or #19 or #20 or #21 | 1,842,998 |
| 23 | #5 and #11 and #15 and #22 | 427 |

6. **Database:** CINAHL

| # | **Query** | **Results from 20 Jan 2026** |
| --- | --- | --- |
| 1 | Caregiver* | 102,051 |
| 2 | Parent* | 237,884 |
| 3 | Mother* | 167,724 |
| 4 | Father* | 52,461 |
| 5 | #1 or #2 or #3 or #4 | 433,260 |
| 6 | Preschool* | 250,050 |
| 7 | Child* | 1,016,002 |
| 8 | Infant* | 364,743 |
| 9 | Toddler* | 9,407 |
| 10 | P#ediatric | 232,036 |
| 11 | #6 or #7 or #8 or #9 or #10 | 1,202,513 |
| 12 | Screen Time | 4,832 |
| 13 | Media exposure | 2,095 |
| 14 | SmartPhone | 13,465 |
| 15 | #12 or #13 or #14 | 19,946 |
| 16 | Training* | 310,479 |
| 17 | Program* | 645,965 |
| 18 | Intervention* | 675,996 |
| 19 | Education* | 918,270 |
| 20 | Experiment* | 218,810 |
| 21 | Randomi?ed | 392,106 |
| 22 | #16 or #17 or #18 or #19 or #20 or #21 | 2,308,317 |
| 23 | #5 and #11 and #15 and #22 | 1,409 |
